# Factors influencing hospital anxiety and depression among emergency department nurses during the COVID-19 pandemic: A multi-center cross-sectional study

**DOI:** 10.3389/fpsyt.2022.912157

**Published:** 2022-08-03

**Authors:** Naif S. Alzahrani, Abdulaziz Mofdy Almarwani, Saeed A. Asiri, Hanan F. Alharbi, Fahad M. Alhowaymel

**Affiliations:** ^1^Department of Medical Surgical Nursing, College of Nursing, Taibah University, Medina, Saudi Arabia; ^2^Department of Psychiatric Nursing, College of Nursing, Taibah University, Medina, Saudi Arabia; ^3^Department of Medical Surgical Nursing, College of Nursing, King Saud University, Riyadh, Saudi Arabia; ^4^Department of Maternity and Child Health Nursing, College of Nursing, Princess Nourah bint Abdulrahman University, Riyadh, Saudi Arabia; ^5^Department of Nursing, College of Applied Medical Sciences, Shaqra University, Shaqra, Saudi Arabia

**Keywords:** mental health, female nurses, urban area, healthcare, psychology, COVID-19

## Abstract

**Introduction:**

The emergency department (ED) is a highly stressful environment, which exposes nurses to infection. ED nurses handle life-threatening conditions, endure long working hours, and deal with anxious patients and their families.

**Aim:**

This study aimed to examine factors, which may influence anxiety and depression levels among ED nurses during the coronavirus disease 2019 (COVID-19) pandemic.

**Methods:**

A cross-sectional design was used with 251 participants from six hospitals in Saudi Arabia (mean age = 32.7 ± 6.59, range = 21–54 years, 70.5% females). Data were collected using the Hospital Anxiety and Depression Scale (HADS), and the analysis was conducted using structural equation modeling (SEM).

**Results:**

Based on the HADS scores, 29.1 and 25.5% of ED nurses were identified as doubtful cases for depression and anxiety, respectively. Additionally, 34.7 and 43.3% of ED nurses were identified as definite cases for depression and anxiety, respectively. Higher anxiety levels were observed among female nurses, nurses with lower physical activity levels, and nurses who worked in urban areas. Low physical activity levels and more than 6 years of work experience correlated with a higher level of depression. None of the hypothesized paths in the anxiety and depression models were significant, except for two observed variables—namely, work location and physical exercise in the anxiety model and physical exercise in the depression model.

**Conclusion:**

Emergency department nurses expressed high levels of anxiety and depression during the COVID-19 pandemic, which may negatively affect their performance and reduce care quality. Therefore, health care leaders should implement specialized mental health education programs focused on nursing occupational safety and support to improve ED nurses’ psychological well-being. Specific attention should be paid to ED female nurses who work in urban areas, especially those with more than 6 years of experience.

## Introduction

The emergency department (ED) is a highly stressful environment, which exposes nurses to infection. ED nurses handle life-threatening conditions, endure long working hours, and deal with anxious patients or their families (1). The spread of coronavirus disease 2019 (COVID-19) and its mutations, such as Omicron, have contributed to increased stress in the ED environment (2, 3). This is because emergency care is the front treatment line for COVID-19 patients. Therefore, ED nurses are more vulnerable to psychological distress, particularly anxiety and depression (2).

Anxiety is a mental health condition characterized by excessive worrying and at least three of the following symptoms: restlessness, fatigue, irritability, difficulty concentrating, muscle tension, or sleep disturbance (3). Depression is a disorder associated with a low mood that impacts an individual’s day-to-day functioning (3). Depression and anxiety symptoms experienced during the pandemic may be associated with individuals’ perception of COVID-19 as a collective traumatic event (4, 5). Both of these conditions are key determinants of psychological distress. They are associated with sleep disturbance, poor coping behaviors such as disordered eating and addictive behaviors, and poor quality of life (6–8).

Before the COVID-19 pandemic, Saudi Arabia and other countries in the region had already experienced outbreaks that negatively impacted the public and healthcare providers. For example, the outbreak of Middle East Respiratory Syndrome Coronavirus (MERS-CoV) was identified in 2012. According to the European Centre for Disease Prevention and Control, 887 reported MERS-CoV cases in 2014, approximately 85% of which were reported in Saudi Arabia (8). Of 386 healthcare providers in Saudi Arabia, where approximately 76% were nurses, a study reported high anxiety levels during this period, and fears of contracting MERS-CoV were high, specifically for professionals working in high-contact areas with suspected or positive virus cases (9). In March 2020, Saudi Arabia declared a national emergency due to the emergence of the COVID-19 pandemic (10), and subsequently, COVID-19’s psychological impact intensified among health care providers. The prevalence of depression, anxiety, and stress among Saudi Arabian healthcare professionals are still high (11). A study conducted in Qatar, during the COVID-19 pandemic, found that 10.6% of healthcare workers tested positive for the virus, and nurses and midwives had the highest infection rates accounting for 33.2% of all the infected healthcare workers (12).

Since the beginning of the COVID-19 outbreak, numerous studies have measured the prevalence of factors contributing to anxiety and depression among healthcare providers. A meta-analysis of 65 studies exploring COVID-19’s psychological impact on healthcare workers reports an anxiety prevalence of 31–38%. This prevalence was higher among nurses than doctors (39.3 vs. 32.5%) (13). Moreover, depression prevalence ranged from 28 to 35%. This prevalence was higher among nurses than doctors (42.4 vs. 39.1%) (13). These high incidences of anxiety and depression are attributed to a variety of factors and characteristics, such as age (14), physical activity (15), work location (16), and years of experience in the medical profession (15, 17, 18). Studies have reported inconsistent figures for the prevalence of anxiety and depression symptoms among nurses. For instance, a cross-sectional national study in China reported that the depression rate was 44% among ED nurses (19). Another recent study during the COVID-19 pandemic found that over half of healthcare providers had anxiety and depression, with nurses reporting higher levels of anxiety and depression than other healthcare providers (20). Conversely, a study in China found lower depression (29%) and anxiety (21%) rates among nurses during the COVID-19 pandemic (21).

The poor psychological health of healthcare providers negatively impacts their personal and professional well-being. At an individual level, poor psychological health could lead to suicide, substance abuse, and physical illnesses such as cardiovascular, musculoskeletal, and metabolic diseases (8, 11, 12). At a professional level, poor psychological health is associated with reduced clinical competency, proneness to clinical errors, communication breakdown, absenteeism, poor job performance, and increased turnover (22). These undesirable consequences of poor psychological health ultimately affect the quality of patient care and safety (22).

Early recognition of psychological health issues among healthcare providers, particularly ED nurses, is essential for improving the quality of patient care and safety. Estimating the prevalence of depression and anxiety among ED nurses is critical for assisting the health authorities in identifying factors contributing to nurses’ distress and implementing mitigation strategies. To our knowledge, there is no data on the prevalence of anxiety and depression among ED nurses in Saudi Arabia during the COVID-19 pandemic. This study aimed to examine the prevalence and influencing factors of hospital anxiety and depression among ED nurses during the COVID-19 pandemic in Saudi Arabia. According to existing literature, several factors could influence hospital anxiety and depression (14–18). Therefore, in this study, we hypothesized that factors including age, sex, marital status, years of experience, work location, shift duration, and physical exercise would influence hospital anxiety and depression levels among ED nurses. We further hypothesized that ED nurses would exhibit high hospital anxiety and depression levels.

## Materials and methods

### Study design

A cross-sectional design was used to examine the factors, which may influence anxiety and depression levels among ED nurses during the COVID-19 pandemic in Saudi Arabia.

### Setting

This study was conducted in two main Saudi Arabian provinces—Medina and Riyadh. Four hospitals in Medina Province were surveyed, with one located outside the city in a non-urban area. Two hospitals in Riyadh Province were surveyed, with one also located outside the city in a non-urban area. Hospitals were chosen based on the level of medical care provided to ensure the inclusion of at least one tertiary and secondary hospital from each province. Moreover, EDs in these hospitals predominantly admitted COVID-19 patients. The data were collected between September and December 2021.

### Sample

**FIGURE 1:**
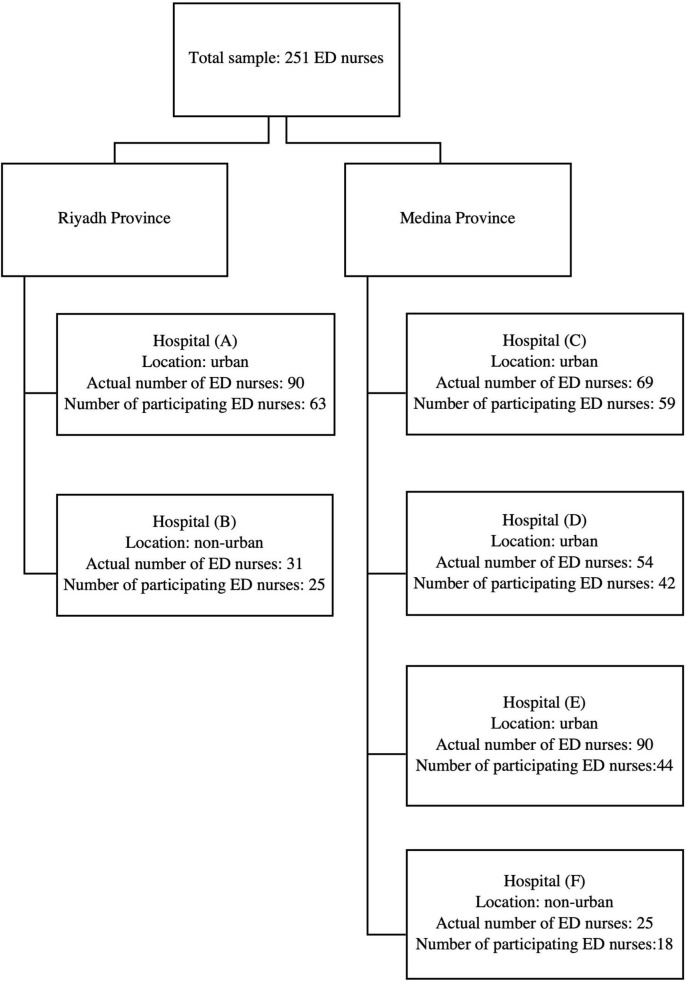
Number of ED nurses approached versus the number of ED nurses who participated.

G*Power was used to determine a sufficient sample size (23) using an alpha of 0.05, a power of 0.80, and a small effect size (*f* = 0.10). The researchers used a small effect size to detect significant results. Based on the aforementioned parameters and small effect size, the desired sample size was 235 participants. An online self-report questionnaire was developed through Google Forms, and the link was sent to head nurses and charge nurses, who distributed the questionnaire among ED nurses. The targeted hospitals had 362 registered ED nurses (Figure 1). In total, 251 questionnaires were completed and submitted, with an overall response rate of 70%. All participants were adults over 18 years old working in EDs as nurses.

### Measurements

Data were collected electronically using an English self-reported questionnaire. The questionnaire comprised two parts. The first part collected demographic data: age, sex, marital status, location of the working hospital, years of experience, physical exercise, and weekly work shifts. The second part included the Hospital Anxiety and Depression Scale (HADS), which is widely used in clinical settings (24). HADS is a 14-item scale with two subscales—seven items to measure anxiety and seven to measure depression. Depression items tend to focus on the anhedonic symptoms of depression, whereas anxiety items tend to focus on generalized anxiety symptoms (25). Each item is scored on a 4-point Likert scale ranging from 0–3. The total scores for both the anxiety and depression subscales range from 0 to 21. A score of 7 or less on the subscales indicates normal anxiety and depression levels, 8–10 indicates doubtful cases of anxiety and depression, and 11–21 indicates a definite case of anxiety and depression (26). Internal reliability coefficients reported in the literature for the scale are quite robust, with a Cronbach’s alpha of 0.83 and 0.82 for the anxiety and depression subscales, respectively (21). In our sample, the HADS exhibited adequate internal consistency with a Cronbach’s alpha of 0.79 and 0.78 for the anxiety and depression subscales, respectively.

### Ethical considerations

Approval for conducting the study was obtained from the Institutional Review Board of the Ministry of Health in Saudi Arabia (150-2021). The researchers did not collect any identifying or personal information from the participants to maintain the latter’s privacy and confidentiality. The primary researcher stored data on a personal computer. Participation in this study was voluntary. Additionally, all participants were made aware of the study’s aims and informed about their right to withdraw at any time.

### Statistical analysis

Statistical Package for the Social Sciences (SPSS) software version 28 and AMOS version 26 were used to analyze the data. Descriptive statistics, including mean, standard deviation, median, IQR, frequency, and percentage, were used to describe the characteristics of the study sample. Additionally, due to the violation of the normality assumption, non-parametric tests (Mann–Whitney U test and Spearman’s rank correlation) were conducted to compare depression and anxiety scores across participants’ factors and examine the relationships among the study variables. Structural equation modeling (SEM) was used to explore the association between these factors. Overall model fit was assessed using the comparative fit index (CFI), standardized root mean square residual (SRMR), and Tucker-Lewis index (TLI). An SRMR <0.08, CFI, and TLI equal to or above 0.95 indicated an adequate model fit (5, 7). We tested the association between anxiety and depression with age, sex, marital status, years of experience, hospital locations, work shifts, and physical exercise factors. A *p*-value of ≤0.05 was considered statistically significant.

## Results

### Demographic characteristics

A total of 251 ED nurses participated in the study. The mean age of the participants was 32.7 years (range = 21–54 years). Most respondents were females (70.5%), married (50.6%), had over 10 years of nursing experience (35.5%), worked an 8-h dayshift (41%), and performed regular physical exercise (57.8%) (Table 1).

**TABLE 1:** Demographic characteristics (*N* = 251).

Measure	*N*	*M*	SD
Age (years)	251	32.7	6.59
**Measure**	***N***	**%**	
Gender			
Male	74	29.5	
Female	177	70.5	
Marital status			
Single	100	39.8	
Married	127	50.6	
Divorced	24	9.6	
Years of experience			
1–3 years	62	24.7	
4–6 years	52	20.7	
7–9 years	48	19.1	
10 years and more	89	35.5	
Work shift per week			
8 h day shift	103	41.0	
8 h evening shift	26	10.4	
8 h night shift	30	12.0	
12 h day shift	48	19.1	
12 h night shift	44	17.5	
Work location			
Urban	224	89.2	
Non-urban	27	10.8	
Do you perform regular physical exercise?			
Yes	145	57.8	
No	106	42.2	

N, number of participants; M, mean; SD, standard deviation; %, percentage.

### Prevalence of anxiety and depression among emergency department nurses and their association with participants’ characteristics

The anxiety score median (Q1–Q3) was 10.0 (7.0–13.0), while the depression score median (Q1–Q3) was 9.0 (6.0–12.0). These scores were divided into three categories: normal scores, doubtful cases, and definite cases. Approximately one-third of ED nurses (36.3%) exhibited normal scores on the depression scale, followed by definite depression cases (34.7%), and finally, doubtful depression cases (29.1%). Simultaneously, most ED nurses (43.4%) were definite cases on the anxiety scale, followed by normal scores (31.1%) and then doubtful cases (25.5%) (Table 2).

**TABLE 2:** Descriptive anxiety and depression statistics among ED nurses (*N* = 251).

Variables	Total score (*N* = 251)	0–7 Normal cases		8–10 Doubtful cases		11–21 Definite cases	
	MD (Q1–Q3)	*N*	%	*N*	%	*N*	%
Prevalence of depression among ED nurses	9.00 (6.00–12.0)	91	36.3	73	29.1	87	34.7
Prevalence of anxiety among ED nurses	10.0 (7.00–13.0)	78	31.1	64	25.5	109	43.4

N, number of participants; MD, median; Q1, first quartile; Q3, third quartile.

Mann–Whitney U test revealed that anxiety scores were significantly higher among female nurses working in ED than male nurses (*U* = 532, *z* = −2.34, *p* = 0.019, with a low effect size *r* = 0.18). ED nurses who performed regular physical exercise reported lower depression and anxiety scores. This difference was significant for both depression (*U* = 595, *z* = −3.05, *p* = 0.024, with a low effect size *r* = 179.19) and anxiety scores (*U* = 640, *z* = −2.25, *p* = 0.002, with a low effect size *r* = 0.14). Anxiety scores were significantly higher among ED nurses working in urban areas than those working in non-urban areas (*U* = 195, *z* = −2.76, *p* = 0.006, with a low effect size *r* = 0.17). Furthermore, nurses with more than 6 years of experience reported higher depression and anxiety scores than nurses with less than 6 years of experience. However, a statistically significant difference was observed in the depression scores (*U* = 660, *z* = −2.11, *p* = 0.035, with a low effect size *r* = 0.13) (Table 3).

**TABLE 3:** Differences in factors contributing to anxiety and depression among ED nurses (*N* = 251).

Variables	*N*	Depression		Anxiety	
		MD (Q1–Q3)	*p*	MD (Q1–Q3)	*p*
Sex			0.869		0.019*
Female	177	9.0 (6.0–11.0)		10.0 (7.0–13.0)	
Male	74	9.0 (5.0–12.0)		9.0 (5.0–12.0)	
Physical activity			0.024**		0.002**
Yes	145	9.0 (6.0–13.0)		9.0 (7.0–14.0)	
No	106	10.0 (5.0–11.0)		10.0 (6.0–12.0)	
Work location			0.179		0.006**
Urban	224	9.0 (6.0–11.7)		10.0 (7.0–13.0)	
Non-urban	26	8.50 (1.0–12.0)		6.00 (1.0–11.2)	
Years of experience			0.035*		0.829
≤6 years	114	9.0 (5.0–11.0)		10.0 (7.0–13.0)	
>6 years	137	10.0 (6.0–12.0)		10.0 (6.0–12.0)	
Work shift duration			0.854		0.367
8 h shift	159	9.00 (6.0–11.0)		10.0 (7.0–12.0)	
12 h shift	92	9.00 (5.0–12.0)		10.0 (6.0–14.00)	
Work shift time			0.759		0.456
Day shift	177	9.00 (5.50–11.0)		10.0 (6.0–13.0)	
Night shift	74	9.00 (5.75–12.0)		10.0 (7.0–13.0)	

*p < 0.05; **p < 0.01. N, number of participants; MD, median; Q1, first quartile; Q3, third quartile.

### The association between participants’ characteristics and hospital depression and anxiety

**FIGURE 2:**
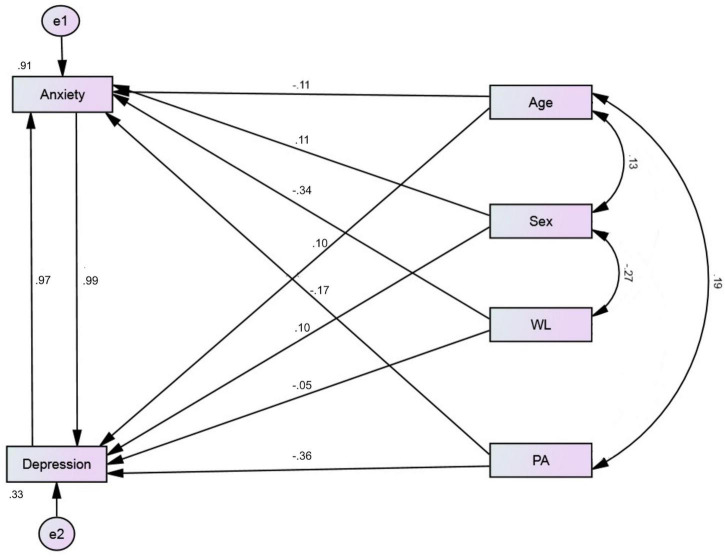
Structural equation path model predicting anxiety and depression in ED nurses.

Table 4 shows a strong correlation between anxiety and depression (*r* = 0.674, *p* = 0.001). Physical activity, work location, age, and sex were weakly correlated with anxiety. Years of experience and physical activity were weakly correlated with depression. Therefore, non-significant variables and paths were not included in the path analysis model, which was used to predict anxiety and depression (Figure 2). This model had an excellent fit for all measures [χ^2^(1) = 0.126, *p* = 0.722, CFI = 1.00, TLI = 1.05, RMSEA = 0.00, SRMR = 0.008], and accounted for 91.5 and 33.0% of the variance in anxiety and depression, respectively. Figure 2 shows that physical exercise negatively predicted depression, whereas anxiety and sex exhibited a significant direct effect on depression. Similarly, depression, physical activity, work location, age, and sex exhibited a significant direct effect on anxiety.

**TABLE 4:** Correlations among anxiety, depression, and participants factors.

Measure	1	2	3	4	5	6	7	8	9
1. Anxiety	–								
2. Depression	0.674**	–							
3. Age	−0.178**	0.062	–						
4. Sex	0.148*	0.010	0.122	–					
5. Marital status	0.037	0.111	0.501**	0.085	–				
6. Work location	−0.175**	–0.085	0.043	−0.267**	–0.029	–			
7. Years of experience	0.038	0.149*	0.729**	0.174**	0.541**	–0.018	–		
8. Physical activity	−0.143*	−0.193**	−0.240**	0.040	0.231**	0.055	0.224**	–	
9. Work shift	0.047	0.019	−0.126*	0.054	–0.050	−0.135*	–0.038	−0.040	–

The symbols * and ** indicate that correlation is significant at the levels of 0.05 and 0.01, respectively.

Physical exercise exhibited a weak indirect effect on depression *via* anxiety (β = −0.161, 95% CI: −3.113 to −0.021, *p* = 0.025). The indirect effects of sex and work location on depression were marginal (*p* = 0.059 and 0.063, respectively). Physical exercise and work location exhibited significant indirect effects *via* depression on anxiety (β = −0.197, 95% CI: −3.113 to −0.021, *p* = 0.004) and (β = 0.202, 95% CI: −0.006 to 2.634, *p* = 0.011), respectively. The indirect effect of age on anxiety was marginal (*p* = 0.064).

## Discussion

This study examined the prevalence of depression and anxiety among ED nurses in Saudi Arabia during the COVID-19 pandemic. The effects of ED nurses’ characteristics on depression and anxiety were also analyzed. Combining respondents in the categories of doubtful and definite cases, approximately 64 and 69% of ED nurses can be reported as cases of anxiety and depression, respectively. These results are similar to a study conducted in China, where researchers found that less than half of 1,103 ED nurses were depressed (19). Anxiety and depression were also high among 441 nurses in Iran (16). In a study conducted in Saudi Arabia during the COVID-19 pandemic, just over half of the 502 healthcare providers reported depressive and generalized anxiety disorders. Nurses exhibited significantly higher depressive and generalized anxiety disorder scores than other healthcare providers (18). Another Saudi Arabian study revealed moderate to high perceived stress among 176 frontline nurses, with high perceived infectability and germ aversion during the COVID-19 pandemic (27). ED nurses often deal with more severe and life-threatening cases that need close-contact interpersonal interactions with infectious patients (28). Moreover, factors such as a heavy workload, adverse events, and erratic working hours contribute to increased depression and anxiety among these nurses (29, 30). Consequently, with the escalation of urgent cases during the COVID-19 pandemic imposing undue strain on an already stressful environment, nurses are likely to experience increased stress and anxiety levels.

In the current study, specific characteristics were associated with the high prevalence of depression and anxiety among ED nurses. Age was a significant negative predictor of anxiety, consistent with several studies that reported higher anxiety levels among young adults during the COVID-19 pandemic (31). Age was negatively correlated with physical activity and positively correlated with marital status and years of experience. We found that regular exercise can significantly reduce the levels of depression and anxiety among ED nurses. This result was expected because regular physical activity tends to reduce the risk of depressive illnesses (32, 33). A study conducted among healthcare workers in Saudi Arabia during the COVID-19 pandemic revealed that inadequate exercise significantly predicted negative mental well-being and low self-efficacy (15).

In the current study, work location only significantly affected ED nurses’ anxiety levels. Nurses who worked in urban areas reported significantly higher anxiety levels than those who worked in non-urban areas. Our results are inconsistent with a study conducted in Iran during the COVID-19 pandemic; wherein there were no significant differences in depression and anxiety between urban and rural areas (16). A possible explanation for this is the large number of patients in our study who visited EDs at hospitals in urban areas, likely owing to the high population density in these areas. These numbers have substantially increased during the COVID-19 pandemic, thus increasing the workload of ED nurses. As mentioned above, the workload is a significant risk factor for negative psychological impacts on ED nurses (29, 30).

The present study’s results further indicated that female nurses were more depressed and anxious than male nurses; however, a statistically significant difference was only observed in anxiety scores. Our results agree with reports from China and Iran during the COVID-19 pandemic, where researchers found that female nurses had significantly higher depression and anxiety scores (16, 21). Our results also align with a study among healthcare providers in Saudi Arabia, which found that female healthcare workers were significantly more depressed and anxious during the COVID-19 pandemic (20). However, our results are inconsistent with a similar study, which found that perceived stress among nurses did not differ by gender during the pandemic (27). In general, women frequently report higher anxiety levels than men (34). This may be due to the prevalence of female nurses with families, whose responsibilities often extend to caring for family members, children, and others. Hence, their fear of contagion may increase while having to attend to ED patients during a pandemic (35).

In the current study, ED nurses with more than 6 years of experience were more depressed and anxious than those with six or fewer years of experience. However, only depression was significantly different between the groups. Our result is inconsistent with studies from Pakistan (17) and Vietnam (18), which showed that nurses with less experience exhibited higher depression and anxiety levels. A study conducted in Saudi Arabia found that healthcare workers with less experience reported negative mental well-being and low self-efficacy (15). The present study’s results were unexpected as most studies indicate that younger nurses experience more negative psychological outcomes than their older counterparts (36–39). However, the COVID-19 pandemic is an anomalous situation and, thus, a possible explanation for the variant results. The pandemic may exacerbate experiences of depression and anxiety among all nurses despite their experience level.

Finally, stress levels were higher among nurses during the COVID-19 pandemic, correlating with higher depression and anxiety levels (40). In the current study, 64 and 69% of ED nurses were classified as doubtful/definite cases of anxiety and depression, respectively. These levels are considered relatively high. A study conducted in Saudi Arabia on 999 international nurses before COVID-19 found slightly lower scores for anxiety and depression compared with our results, where 54% of nurses were depressed and 65% were anxious (41). Another study comprising 102 Australian nurses before the COVID-19 pandemic showed even lower scores, with 32.4 and 41.2% of nurses being depressed and anxious, respectively (42). The prevalence of anxiety and depression among 850 nurses in a Hong Kong study was also low (37.3 and 35.8%, respectively) (43). Therefore, we concluded that ED nurses experienced substantially higher levels of anxiety and depression during the COVID-19 pandemic.

### Limitations

This study has some noteworthy limitations. The first is using a cross-sectional design, which only provides a snapshot of the participants at a given time. The second is using a convenience sampling method with a small sample size, which could result in respondent bias due to the group’s heterogeneity, such as in the age and gender of participants. This could reduce the results’ generalizability. The third limitation is using an English version of the measurement with nurses from different backgrounds. Although all the nurses understood the language, this could have affected the accuracy of anxiety and depression measurements, as English is not their first language. Lastly, our results support the protective role of physical exercise. However, details on the type, frequency, and duration of physical activity performed were not assessed. This point should be addressed in future studies in order to maximize the use of exercise as a distress-mitigating intervention among ED nurses.

## Conclusion

The results of this study demonstrate a moderate-to-high prevalence of anxiety and depression among ED nurses in Saudi Arabia during the COVID-19 pandemic, with certain demographic characteristics associated with this high prevalence. Higher anxiety levels were associated with being female, low levels of physical activity, and working in an urban area. Similarly, low levels of physical activity and having more than 6 years of experience were correlated with a high level of depression. In conclusion, ED nurses in Saudi Arabia might suffer from psychological distress, particularly anxiety and depression, which could impact their performance and reduce the quality of care. Therefore, healthcare leaders in Saudi Arabia should implement specialized mental health education programs focused on nursing occupational safety and support. These programs can help improve ED nurses’ psychological well-being. Specific attention should be paid to ED female nurses who work in urban areas, especially those with more than 6 years of experience. ED nurses must be involved in stress management and coping strategy programs to maintain psychological well-being and reduce psychiatric comorbidities.

## Data availability statement

The raw data supporting the conclusions of this article will be made available by the authors, without undue reservation.

## Ethics statement

The studies involving human participants were reviewed and approved by the Saudi Ministry of Health Institutional Review Board. The patients/participants provided their written informed consent to participate in this study.

## Author contributions

NA: conceptualization, methodology, formal analysis, and writing—original draft, review and editing. AA and FA: formal analysis, writing—original draft, review and editing. SA and HA: data curation, and writing—review and editing. All authors contributed to the article and approved the submitted version.
